# MicroRNA-1 Inhibits the Growth of Breast Cancer Cells MDA-MB-231 and MCF-7 Treated with Hydatid Cyst Fluid

**DOI:** 10.1155/2024/7474039

**Published:** 2024-03-12

**Authors:** Hadis Jafari, Mahmoud Mahami-Oskouei, Adel Spotin, Behzad Baradaran, Dariush Shanehbandi, Amir Baghbanzadeh, Zahra Alizadeh

**Affiliations:** ^1^Immunology Research Center, Tabriz University of Medical Sciences, Tabriz, Iran; ^2^Department of Parasitology and Mycology, Faculty of Medicine, Tabriz University of Medical Sciences, Tabriz, Iran; ^3^Department of Parasitology and Mycology, School of Public Health, Tehran University of Medical Sciences, Tehran, Iran

## Abstract

Antigens in hydatid cyst fluid (HCF) have been discovered to bear a significant resemblance to antigens present in cancer cells. MicroRNA-1 (miR-1) is a well-known member of the tumor inhibitor miRNA family and has been shown to have pro-apoptotic and tumor-inhibitory functions. This study aimed to evaluate the ability of HCF to prevent breast cancer and to explore the underlying mechanisms that affect cancer cells. For this study, MDA-MB-231 and MCF-7 breast cancer cells were cultured and divided into two groups: one group received HCF treatment and the other group was untreated and served as the control group. The cytotoxicity and cell viability of various HCF concentrations on breast cancer cells were evaluated using the MTT assay. In addition, the expression level of miR-1 in HCF-treated and untreated breast cancer cells was analyzed using qRT-PCR. The study found that HCF treatment reduced the growth of MDA-MB-231 and MCF-7 breast cancer cells, indicating that it was cytotoxic to the cells. Specifically, the IC50 concentration of HCF after 24 hours of treatment was 7.32 *µ*g/mL for MDA-MB-231 cells and 13.63 *µ*g/mL for MCF-7 cells. In addition, qRT-PCR analysis revealed that the expression level of miR-1 was significantly increased in HCF-treated MDA-MB-231 (*P*=0.0203) and MCF-7 (*P*=0.0394) cell lines compared to untreated controls. Although HCF has been shown to inhibit the growth of breast cancer cells and to upregulate miR-1, a key tumor suppressor in cancer cells, the specific mechanisms responsible for this effect remain unclear. Further studies are needed to fully understand the molecular pathways underlying HCF's antitumor activity and its potential as a therapeutic agent in cancer therapy.

## 1. Introduction

Hydatid cysts, the larval stage of *Echinococcus granulosus*, can develop anywhere in the body [1]. The cyst consists of three layers that are surrounded by a layer produced by the host. The outer layer is fibrous, while the laminated membrane, also referred to as the cuticle or hyaline layer, is multilayered and acts as a protective cyst. The germinal membrane is a very thin and granular layer on the surface where brood capsules and protoscolices are produced. The outer laminated and inner germinal layers are filled with HCF [1, 2]. Some studies have suggested a relationship between hydatid cysts and cancers [3–6], while the anticancer effects of parasites such as *Trypanosoma cruzi*, *Toxoplasma gondii,* and *Trichinella spiralis* have also been investigated [7–9]. Breast cancer, a common malignancy in women, is closely related to the expression of oestrogen/progesterone receptors [10, 11]. Breast cancer treatment typically involves radiotherapy, mastectomy, and anticancer drugs such as doxorubicin (DOX), docetaxel (DTX), and paclitaxel (PTX). However, developing novel cancer therapies is challenging for oncology researchers due to a range of limitations and side effects [12, 13]. Several studies have demonstrated that hydatid cysts are associated with the inhibition of tumor cell proliferation, although which components of the cysts induce this effect remain to be fully understood [3, 10]. Although there is extensive literature on the involvement of parasites in various types of cancer, the role of *Echinococcus granulosus* has largely remained unexplored [14].

MicroRNAs (miRNAs) are small, noncoding RNAs that induce RNA silencing and regulate gene expression [15]. These molecular structures regulate both physiological and pathological cellular processes, and many of them can function as either oncogenes or tumor suppressors [16]. MiR-1 is one of the most well-known members of the tumor-suppressive miRNA family, which has pro-apoptotic and tumor-inhibitory functions [17]. This miRNA inhibits the proliferation and differentiation of cancer cells and increases apoptosis in cancer cells [17, 18]. Various studies have shown that miR-1 has inhibitory effects on cancers including breast, liver, ovarian, and esophageal cancer [17–19]. In breast cancer, miR-1 targets two genes, K-RAS and MALAT1, to reduce proliferation and differentiation and induce cancer apoptosis [20].

We evaluated the above hypothesis regarding the effects of HCF (known for its anticancer properties) on MDA-MB-231 and MCF-7 breast cancer cells and investigated the mechanism by which this effect occurs in cancer cells.

## 2. Materials and Methods

### 2.1. Hydatid Cyst Fluid

*E. granulosus* cysts were collected from a slaughterhouse in East Azerbaijan, northwest of Iran. HCF was extracted by aspiration, centrifuged (800 g for 15 min), filtered, and then stored.

### 2.2. Cell Lines

MDA-MB-231 and MCF-7 cell lines from the American Type Culture Collection (ATCC) of the National Cell Bank (Institute Pasteur, Iran) were used in this study. Cells were cultured in IMDM (Invitrogen, USA) and treated with 10% FBS (Invitrogen) at 37°C and 5% CO_2_.

### 2.3. MTT Cell Viability Assay

IC50 (half-maximal inhibitory concentration) of HCF on the cell lines was determined using the MTT assay. First, 8 × 10^5^ cells of MDA-MB-231 and 8 × 10^3^ cells of MCF‐7 were seeded in a 96-well plate, and 200 *µ*L of medium was added to each well and cultured for 24 hours. Then, the medium was removed and followed by adding various concentrations of HCF (5–100 *µ*g/mL) and 200 *μ*l of fresh medium, and incubation at 37°C for 24 hours with 5% CO_2_. Phosphate-buffered saline (PBS) was applied to remove the culture medium, and 150 *μ*l medium and 2 mg/mL MTT solution (50 *μ*l; Sigma, USA) were transferred to the wells, and incubation was carried out for 4 hours at 37°C and 5% CO_2_. After removing the supernatant, Sorenson's buffer and dimethyl sulfoxide (DMSO) were added to the wells, which were then returned to the incubator under dark conditions. To determine the IC50 of HCF for both cell lines, the optical density of each well was read separately at 570 nm after a 30-minute incubation period.

### 2.4. Cell Treatment

8 × 10^5^ MDA-MB-231 and 8 × 10^3^ MCF‐7 were seeded in a 6-well plate and then incubated overnight at 37°C, 5% CO_2_. After the addition of HCF at IC50 concentration (obtained by MTT assay) to the wells, incubation was carried out for 6, 12, and 24 hours.

### 2.5. RNA Extraction

After culturing and treating the cells, RNA extraction was performed using the RiboExTM protocol developed by GeneAll Biotechnology (South Korea). To confirm the concentration and purity of the extracted RNAs, electrophoresis was performed on a 1.5% agarose gel, and the optical density at A260 and A280 was calculated using a Nanodrop spectrophotometer from Thermo Scientific (USA).

### 2.6. Complementary DNA (cDNA) Synthesis and Real-Time Polymerase Chain Reaction (RT-PCR)

The miRCURY LNA™ Universal cDNA Synthesis Kit II (Exiqon, Vedbaek, Denmark) was used to prepare cDNA. RT-PCR was carried out using SYBR Green PCR Master Mix (YectaTajhizAzma, Iran) and miRNA-LNA™ PCR primers at 200 nM concentration to measure miR-1 expression in MDA-MB-231 and MCF-7 cells (Table 1). The SYBR Green RT-PCR conditions were as follows: 10 minutes at 95°C, 40 cycles 10 seconds at 95°C (denaturation), and 30 seconds at 60°C (annealing). The nontemplate control was prepared using nuclease-free water. The relative miR-1 expression of untreated and HCF-treated breast cancer cells was analyzed using the Ct (2–^ΔΔCt^) formula. The internal control for this analysis was the expression level of the U6 snRNA. The values of the treated breast cancer cell lines were compared to those of the untreated ones.

### 2.7. Statistical Analysis

The miRNA gene expression levels were measured as fold changes between the HCF-treated and untreated breast cancer cells, and the measurements were performed three times. GraphPad Prism 6.0 was used for data analysis. Student's *t*-test was used for significant differences, and *p* value <0.05 was considered significant.

## 3. Results

### 3.1. Growth of Breast Cancer Cells in IMDM

**Figure 1:**
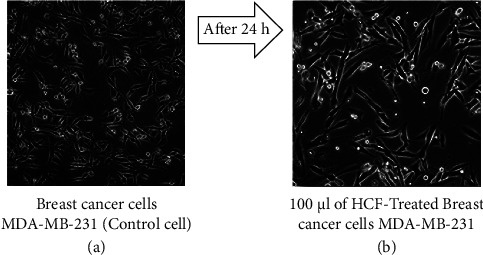
The morphology evaluation of control and HCF-treated MDA-MB-231 cells (a, b).

**Figure 2:**
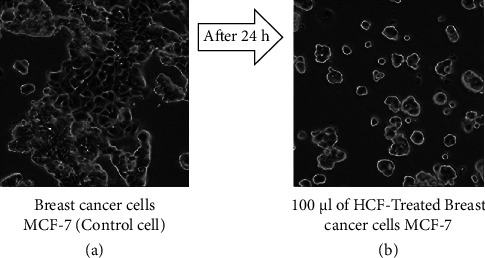
The morphology evaluation of control and HCF-treated MCF-7 cells (a, b).

The present study examined the growth of MDA-MB-231 and MCF-7 cells in IMDM containing 10% FBS. Figures 1(a) and 2(a) show microscopic images of cultured MDA-MB-231 and MCF-7  cells, respectively.

### 3.2. Anticancer Effects of HCF in Culture Model

**Figure 3:**
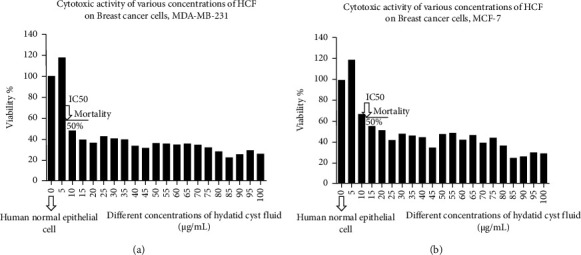
The cytotoxic effect of different concentrations of HCF-treated breast cancer cells. (a) MDA-MB-231. (b) MCF-7, at 570 nm by an MTT assay following the exposure time of 24 h. The HCF IC50 value on MDA-MB-231 and MCF-7 cells was 7.32 and 13.63 *μ*g/mL, respectively.

Treatment of MDA-MB-231 and MCF-7 cells with different concentrations of HCF (5–100 *μ*g/mL) for 24 hours resulted in 50% cell inhibition. The IC50 values were determined for both cell types, reflecting the potency of HCF examined in the MTT assay between the control and treatment groups. The viability of HCF-treated cells was compared to untreated MDA-MB-231 and MCF-7 cells (control group). The IC50 concentration of HCF after 24 hours of treatment was 7.32 and 13.63 *μ*g/mL for MDA-MB-231 and MCF-7, respectively. No significant effect on cell proliferation was observed when 5 *μ*g/mL HCF was incubated with breast cancer cells. However, when higher HCF concentrations were used, cell proliferation was inhibited and cell lysis increased in MDA-MB-231 and MCF-7 cells (Figures 3(a) and 3(b)). The cytotoxicity of HCF was evaluated by observing the morphology of the cells under a microscope after treatment with the IC50 concentration of HCF. The images (Figures 1(b) and 2(b)) showed a reduction in cell number and structural alterations in the treated cells, such as cell shrinkage, altered membrane integrity, and inhibited cell growth.

### 3.3. Fold Change in MiR-1 Expression in HCF-Treated MDA-MB-231 and MCF-7 Cells

**Figure 4:**
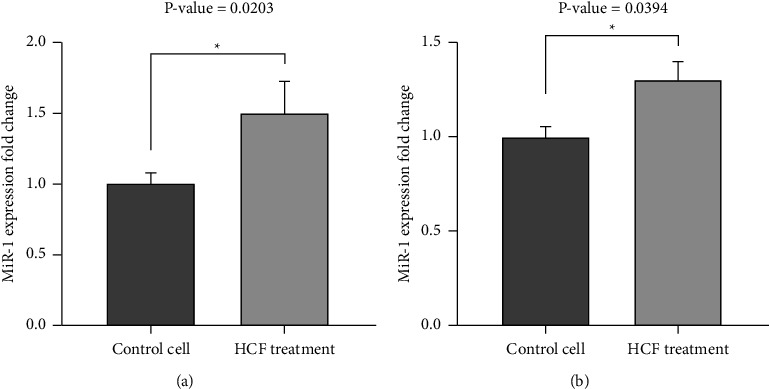
The miR-1 expression level in breast cancer cell lines treated with hydatid cyst fluid (HCF) compared to control cells. (a) MDA‐MB‐231, (b) MCF‐7.

Quantitative PCR (qPCR) was used to evaluate the expression of miR-1. According to the results, the expression level of miR-1 increased in both MDA-MB-231 and MCF-7 cells following treatment with HCF. MiR-1 expression level increased in MDA-MB-231 with an approximately 1.5-fold change (*P*=0.0203) and in MCF-7 with an approximately 1.4-fold change (*P*=0.0394) after receiving HCF (Figures 4(a) and 4(b)).

## 4. Discussion

Cancer as a global health problem is one of the leading causes of death in this century. Based on the latest data from the American Cancer Society, 1,918,030 new cases and 609,360 deaths were recorded in 2022. These statistics highlight the urgent need for continued research and development of effective treatments and prevention strategies for cancer [21]. Breast cancer, which is known as the most common cancer among women worldwide, is one of the leading causes of cancer-related death [22]. The disease is characterized by the abnormal growth and division of cells, leading to uncontrolled proliferation and disruption of the apoptotic process, which ultimately results in cancer development. Unfortunately, there is no single definitive treatment for breast cancer, which makes early detection and timely treatment all the more crucial in reducing mortality rates and improving patient outcomes [11, 22]. The complex and diverse nature of cancer, including breast cancer, presents a major challenge in defining it as a single and static entity. Cancer arises from cells with different biological, cytological, and pathological characteristics [23]. Breast cancer can be treated using various approaches, including surgery, and chemotherapy. However, newer therapies are being developed that aim to be more effective and have fewer side effects. These new therapies hold great promise in the fight against breast cancer. The development of these newer therapies and ongoing research efforts to uncover the underlying mechanisms of cancer will be vital in improving patient outcomes and reducing the devastating impact of breast cancer [23, 24].

Previous studies have suggested that parasites could potentially have an anticancer effect. For instance, in 2011, Chen et al. demonstrated in a mouse model that malaria infection significantly suppressed the growth of Lewis lung carcinoma (LLC) by stimulating adaptive and innate antitumor responses [25]. Another study found that *Trypanosoma cruzi* calreticulin had antiangiogenic and antitumor properties [7]. Additionally, the *Toxoplasma* lysate antigen (TLA) was found to have antitumor effects on human glioma U373MG and U87MG cells both in vitro and in vivo [26]. *Trichostrongylus colubriformis* excretory/secretory products inhibit the growth of ovarian epithelial cells and fibroblasts (3T3) [27]. More recently, it has been demonstrated that hydatid cyst protoscoleces can suppress tumor growth, indicating potential antitumor properties of these parasites [3–6]. The aim of this study was to evaluate the anticancer effect of HCF on breast cancer cells, specifically MDA-MB-231 and MCF-7, in vitro. This study represents a successful attempt to use the antitumor effects of HCF to control the growth of these cells in vitro. The ability of HCF to prevent cancer has also been demonstrated in previous studies, as evidenced by a study that found HCF inhibits the growth of melanoma cells [28]. Furthermore, mucin-like peptides (Egmuc peptides) derived from *Echinococcus granulosus* have been shown to have antitumor effects [29]. Another study by Berriel et al. in 2013 reported that human HCF had antitumor activity in an animal model of colon carcinoma [3].

MiRNAs are involved in many different cell functions as part of gene regulation. Any abnormalities in the cell, such as the onset and progression of cancer, can lead to changes in miRNA expression [30]. The significance of miRNAs in cancer was first demonstrated in chronic lymphocytic leukemia [31], and subsequent investigations have shown their involvement in the development of different diseases such as parasitic infections, neurological disorders, cancer, and cardiovascular diseases [32–34]. Due to their involvement in the development, progression, and invasion of cancers, any changes in miRNA expression have been proposed as biomarkers for diagnosis and treatment [35]. One of the most well-known miRNA families associated with tumor suppression is miR-1, which has pro-apoptotic and differentiation-inhibiting functions [16, 17].

Numerous researches have revealed that miR-1 plays a crucial role in suppressing the growth and differentiation processes of cancer cells and promoting apoptosis [17–19]. These findings suggest that miRNA-based therapeutics may be a hopeful approach to cancer therapy. MiR-1 can suppress the growth of ovarian cancer cells by targeting p-Akt, p-ERK1/2, p-Rb, and CDK4 in HO-8910PM cells and suppress c-Met expression, which shows its inhibitory effect [17]. Zhang et al. reported that increasing miR-1 expression in liver cancer cells led to apoptosis in HCCLM3 and Bel-7474 cell lines and inhibited cancer cell growth by targeting the SOX9 gene and reducing its expression [18]. Additionally, a study found that miR-1 can impede the growth of esophageal squamous cell carcinoma by downregulating the expression of MET, CDK4, and cyclin D1 [19].

This study investigated changes in miR-1 expression in HCF-treated breast cancer cells and found decreased cancer cell numbers and altered patterns. These results suggest that HCF has an anticancer effect by regulating miR-1, which could be an important anticancer and therapeutic target for breast cancer. Further investigations will be necessary to refine our understanding of target selection for treatment candidates in breast cancer.

## 5. Conclusion

Upregulation of miR-1 in cancer cells treated with HCF can inhibit MDA-MB-231 and MCF-7 growth and decrease cell motility. These findings provide evidence for the crucial role of miR-1 in the proliferation of breast cancer cells and indicate that miR-1 and HCF may have potential prognostic and therapeutic applications. However, the mechanisms underlying the functions of miR-1 via target genes in breast cancer remain unclear, and further studies are recommended to better understand the molecular mechanisms involved.

## Figures and Tables

**Table 1:** Primer sequences and annealing temperatures applied in RT-PCR.

Name	Sequence(s)	Annealing temperature
MiR-1	5′-GGTGCGGTGACATACTTCTTT-3′	60°C
U6	5′-GCTTCGGCAGCACATATACTAAAAT-3′	60°C

## Data Availability

The data used to support the findings of this study are included within the article.
